# Fluid management in critically ill patients: the role of extravascular lung water, abdominal hypertension, capillary leak, and fluid balance

**DOI:** 10.1186/2110-5820-2-S1-S1

**Published:** 2012-07-05

**Authors:** Colin Cordemans, Inneke De laet, Niels Van Regenmortel, Karen Schoonheydt, Hilde Dits, Wolfgang Huber, Manu LNG Malbrain

**Affiliations:** 1 Department of Intensive Care, Ziekenhuis Netwerk Antwerpen, Campus ZNA Stuivenberg, Lange Beeldekensstraat 267, 2060 Antwerpen 6, Belgium; 2 II. Medizinische Klinik, Klinikum Rechts der Isar, Technische Universität München, Munich, Germany

**Keywords:** abdominal pressure, extravascular lung water, fluid balance, fluid management, capillary leak, organ failure, prognosis

## Abstract

****Introduction**:**

Capillary leak in critically ill patients leads to interstitial edema. Fluid overload is independently associated with poor prognosis. Bedside measurement of intra-abdominal pressure (IAP), extravascular lung water index (EVLWI), fluid balance, and capillary leak index (CLI) may provide a valuable prognostic tool in mechanically ventilated patients.

**Methods:**

We performed an observational study of 123 mechanically ventilated patients with extended hemodynamic monitoring, analyzing process-of-care variables for the first week of ICU admission. The primary outcome parameter was 28-day mortality. Δ_max_EVLWI indicated the maximum difference between EVLWI measurements during ICU stay. Patients with a Δ_max_EVLWI <−2 mL/kg were called 'responders'. CLI was defined as C-reactive protein (milligrams per deciliter) over albumin (grams per liter) ratio and conservative late fluid management (CLFM) as even-to-negative fluid balance on at least two consecutive days.

****Results**:**

CLI had a biphasic course. Δ_max_EVLWI was lower if CLFM was achieved and in survivors (−2.4 ± 4.8 vs 1.0 ± 5.5 mL/kg, *p *= 0.001; −3.3 ± 3.8 vs 2.5 ± 5.3 mL/kg, *p *= 0.001, respectively). No CLFM achievement was associated with increased CLI and IAP_mean _on day 3 and higher risk to be nonresponder (odds ratio (OR) 2.76, *p *= 0.046; OR 1.28, *p *= 0.011; OR 5.52, *p *= 0.001, respectively). Responders had more ventilator-free days during the first week (2.5 ± 2.3 vs 1.5 ± 2.3, *p *= 0.023). Not achieving CLFM and being nonresponder were strong independent predictors of mortality (OR 9.34, *p *= 0.001 and OR 7.14, *p *= 0.001, respectively).

****Conclusion**:**

There seems to be an important correlation between CLI, EVLWI kinetics, IAP, and fluid balance in mechanically ventilated patients, associated with organ dysfunction and poor prognosis. In this context, we introduce the global increased permeability syndrome.

## Introduction

Acute inflammatory injury incites a cascade of proinflammatory mediators leading to microcirculatory dysfunction, capillary leak, and distributive shock [1,2]. Although in the early stage of shock liberal and goal-directed fluid therapy is mandated [3], subsequent (over)resuscitation increases microvascular hydrostatic pressure and may promote interstitial fluid accumulation [4-6]. This fluid overload is independently associated with impaired organ function, intra-abdominal hypertension (IAH), and poorer outcome [7-15]. Conversely, a conservative fluid strategy limiting fluid intake and even promoting fluid removal improved clinical outcomes [16].

As the lungs are maximally exposed to the proinflammatory cascade, receiving the entire cardiac output, they provide valuable insight into dynamic microcirculatory changes during systemic inflammation [17]. Consequently, bedside measurement of extravascular lung water index (EVLWI) performed by single transpulmonary thermodilution allows the estimation of the extent of capillary leak and fluid overload [11,18-23]. In this study, we investigated the prognostic value of EVLWI, capillary leak parameters, IAH, and fluid balance in critically ill patients.

## Methods

### Patients

We collected data from March 2004 to August 2007 in 123 patients treated in two ICU's in Ziekenhuis Netwerk Antwerpen (ZNA) Campus ZNA Stuivenberg, Antwerp, Belgium. Critically ill patients requiring mechanical ventilation (MV) and, according to clinical appraisal, extended hemodynamic monitoring by single transpulmonary thermodilution technique were consecutively included. Internal review board approval was obtained, and due to the non-interventional and retrospective nature of the study, the need for informed consent was waived (EC approval number 3765).

### Definitions

*Acute lung injury *(*ALI*) and acute respiratory distress syndrome (*ARDS*) were diagnosed according to international criteria [24].

*EVLWI *was recorded as the mean of two daily EVLWI measurements. EVLWI_min,max,mean _were the minimal, maximal, and mean EVLWI during ICU stay, respectively. Maximum EVLWI was measured on Day_max_. Δ_max_EVLWI indicated the maximum difference between all EVLWI measurements during ICU stay and was computed in accordance with overall EVLWI trend (ΔEVLWI or the difference between the first and the last recorded EVLWI). If during ICU stay an increase of EVLWI was recorded followed by an equal EVLWI drop, Δ_max_EVLWI was given the sign of ΔEVLWI. Patients with an EVLWI decrease of >2 mL/kg (Δ_max_EVLWI <−2 mL/kg) and an overall drop in EVLWI during the first week of ICU admission (negative ΔEVLWI) were called 'responders'.

*Intra-abdominal pressure *(*IAP*) was the mean of two daily IAP measurements. IAP_max,mean _were the maximum and the mean IAP during ICU stay. IAH was defined as IAP_mean _≥ 12 mmHg and abdominal perfusion pressure (APP) as mean arterial pressure (MAP) minus IAP according to consensus definitions [15].

Daily *fluid balance *was calculated by subtracting the urinary output from the fluid intake (including both IV and enteral fluid administration); each day cumulative fluid balance was computed by the addition of daily fluid balances.

*Capillary leak index *(*CLI*) was defined as C-reactive protein (CRP) (milligrams per deciliter) over albumin (grams per liter) ratio, multiplied by 100 [25].

*Conservative late fluid management *(*CLFM*) was determined as even-to-negative fluid balance on at least two consecutive days during the first week of ICU stay [12]. In this study, CLFM was used as a descriptive term and did not signify any study intervention.

### Data collection and methods

For the entire duration of the ICU stay, relevant demographic, clinical, and laboratory data along with daily assessment of fluid balance, sequential organ failure assessment (SOFA) score [26], IAP, MV settings, and extended hemodynamic monitoring variables were registered in an electronic database, supplemented by mortality on day 28. Severity of illness on ICU admission was described by an averaged simplified acute physiology score [27] and acute physiology and chronic health evaluation score [28].

IAP was measured via a Foley bladder catheter as described previously [29], in the complete supine position and in stable conditions twice daily. In patients with IAH, the IAP was also continuously monitored via a balloon-tipped catheter placed in the stomach connected to the CiMON monitor (Pulsion Medical Systems, Munich, Germany).

A central venous catheter and a thermistor-tipped arterial thermodilution catheter (Pulsiocath 5F) inserted into the femoral artery and attached to a PiCCOplus^® ^system (Pulsion Medical Systems, Munich, Germany) were already in place for each patient. Transpulmonary thermodilution measurements were obtained by central venous injection of three 20-mL boluses of cooled saline (<8°C). For each set of thermodilution determinations, the mean values were used for statistical analysis. Cardiac output (CO), global end diastolic volume (GEDV), extravascular lung water (EVLW), global ejection fraction (GEF), pulmonary vascular permeability index (PVPI), stroke volume variation, and pulse pressure variation were calculated using the PiCCOplus^® ^[18]. EVLW was indexed to body weight (EVLWI) and CO and GEDV to body surface area (cardiac index, GEDV index).

### Study design

In this observational study, no protocol-directed intervention was performed; treatment was based on recent ICU guidelines. We analyzed process-of-care variables for the first 7 days of ICU admission. The primary outcome parameter was 28-day mortality. Secondary outcome parameters were organ dysfunction, duration of MV, and achievement of CLFM.

### Statistical analysis

The primary data analysis compared survivors to nonsurvivors according to 28-day mortality. Subsequently, patients were stratified by occurrence of IAH, achievement of CLFM, and responders vs nonresponders.

Continuous data were expressed by mean ± SD, and intergroup differences were determined by one-way analysis of variance (ANOVA) analyses day by day for 1 week. Categorical data were expressed as frequency distributions and/or percentages, and the *χ*^2 ^test was used to determine intergroup differences. Two-sided *p *values <0.05 were considered to indicate statistical significance.

Time course of CLI, total SOFA score, EVLWI, APP, daily, and cumulative fluid balance was described by clustered error bar graphs representing mean ± SE. Receiver-operating characteristic (ROC) curves were determined and optimal cutoffs for CLI, EVLWI, and Δ_max_EVLWI were derived, creating categorical data.

Stepwise multivariate logistic regression was performed to determine the independent risk factors for 28-day mortality and for not achieving CLFM. Risk factors significant at the 0.1 level in univariate analysis were included in the models. The Hosmer-Lemeshow test was used assessing the goodness of fit.

The Kaplan-Meier method was used to analyze differences in cumulative survival and duration of MV; distribution was compared using the log-rank test. We used SPSS software package (version 17.0.1; SPSS, Chicago, IL, USA) for data analysis.

## Results

### Patients

We included 123 predominantly medical (*n *= 109) patients on MV, of whom 65 (53%) died after 28 days. At baseline, no significant differences were found between groups, as shown in Table 1, except for lower MAP and GEF in nonsurvivors.

**Table 1:** Baseline characteristics

Variable	Survivors (*n *= 58)	Nonsurvivors (*n *= 65)	*p *value
Age (years)	63.2 ± 14.2	65.3 ± 15.2	0.436

Male sex (%)	66	67	0.798

BMI (kg/m2)	26.6 ± 6.6	24.6 ± 4.0	0.053

Primary reason for MV (%)			0.937
Sepsis/septic shock	24.1	24.7	
Pneumonia	15.5	16.9	
ARDS	13.7	10.8	
Postoperative/trauma	5.4	6.1	
Acute COPD exacerbation	6.9	7.7	
Congestive heart failure	6.9	6.2	
Cardiac arrest	5.2	6.2	
Hemorrhagic stroke	8.6	7.6	
Other	13.7	13.8	

Medical ICU (%)	40.7	48.0	0.562

ICU stay (day)	31.8 ± 18.1	11.0 ± 6.4	<0.001

Severity of disease			
SAPS II	49.5 ± 15.6	53.9 ± 18.1	0.157
APACHE II	22.1 ± 8.5	23.0 ± 10.7	0.617
SOFA score at admission	10.3 ± 4.3	10.4 ± 4.5	0.844

Acute lung injury (%)			0.836
Primary	27.5	30.7	
Secondary	25.8	21.5	

Organ function assessment			
Number of organs failing	2.2 ± 1.3	2.1 ± 1.2	0.605

Hemodynamic variables			
HR (bpm)	96.7 ± 20.4	98.6 ± 18.8	0.661
Mean arterial pressure (mmHg)	84.2 ± 13.4	78.7 ± 10.4	0.011
Met shock criteria (%)	69.0	69.2	0.975
Vasopressor use (%)	69.0	67.8	0.880
CI (L/min/m^2^)	3.6 ± 1.1	3.1 ± 1.6	0.255
SVV (%)	11.8 ± 7.1	14.4 ± 6.9	0.236
GEF (%)	21.2 ± 8.1	15.1 ± 7.7	0.015
GEDVI (mL/m^2^)	766.2 ± 165.0	725.6 ± 174.5	0.42
EVLWI (mL/kg)	9.8 ± 3.9	10.5 ± 5.2	0.543
PVPI	2.4 ± 0.9	2.4 ± 1.1	0.869

Respiratory variables			
Tidal volume (mL/kg of PBW)	8.2 ± 1.7	8.3 ± 1.7	0.709
Plateau pressure (cmH_2_O)	23.8 ± 6.5	24.1 ± 8.2	0.792
PEEP (cmH_2_O)	7.0 ± 2.2	6.2 ± 2.3	0.075
Dynamic compliance (mL/cmH_2_O)	40.9 ± 15.6	39.0 ± 22.6	0.635
PaO_2_/FIO_2_	263.4 ± 135.1	271.7 ± 154.9	0.755

Renal and metabolic variables			
Creatinine (mg/dL)	1.8 ± 1.7	2.5 ± 2.9	0.095
Urine output (mL/day)	1,524.6 ± 1,342.7	1,428.5 ± 1,236.6	0.683
Albumin (mg/dL)	25.0 ± 7.5	27.0 ± 8.7	0.194
pH	7.35 ± 0.11	7.32 ± 0.12	0.205

Immune system			
CRP (mg/dL)	10.6 ± 9.8	13.8 ± 12.4	0.127
Central nervous system			
Glasgow Coma Score	8.1 ± 5.1	8.2 ± 5.2	0.905

Capillary leak index	52.7 ± 56.2	61.8 ± 61.3	0.411

Intra-abdominal pressure (mmHg)	8.2 ± 3.5	7.9 ± 3.7	0.722

Abdominal perfusion pressure (mmHg)	75.4 ± 13.9	70.4 ± 11.3	0.071

Fluid balance (mL/day)	1,755.9 ± 4,616.0	2,133.8 ± 3,525.1	0.612

BMI, body mass index; MV, mechanical ventilation; ARDS, acute respiratory distress syndrome; COPD, chronic obstructive pulmonary disease; SAPS, simplified acute physiology score; APACHE, acute physiology and chronic health evaluation; SOFA, sequential organ failure assessment; HR, heart rate; CI, cardiac index; SVV, stroke volume variation; GEF, global ejection fraction; GEDVI, global end-diastolic volume index; EVLWI, extravascular lung water index; PVPI, pulmonary vascular permeability index; PBW, predicted body weight; PEEP, positive end-expiratory pressure; CRP, C-reactive protein.

### Process-of-care variables

**Figure 1:**
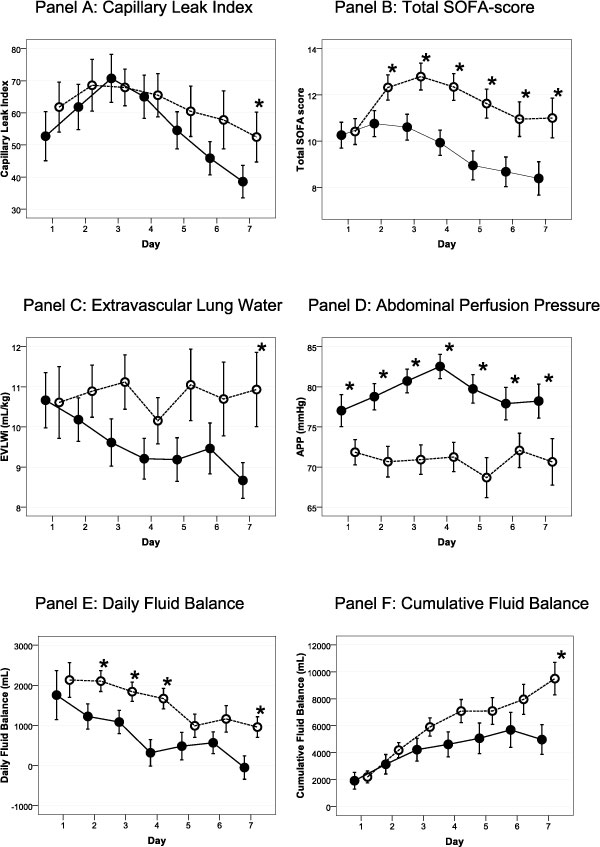
**Time course of main variables**. Mean ± standard error of pertinent variables for the first week after ICU admission. Survivors are depicted by a full line and nonsurvivors by a dotted line. *p < 0.05, day-by-day pairwise compared between survivors and nonsurvivors (one-way ANOVA).

Figure 1 depicts process-of-care variables stratifying patients by survival.

#### CLI

CLI had a biphasic course with a maximum on day 3, which was significantly higher in patients not achieving CLFM (76.1 ± 49.6 vs 53.2 ± 45.6, *p *= 0.017). ROC statistics for CLI on day 3 to predict no CLFM achievement revealed an area under the curve (AUC) of 0.658 and a derived cutoff point of >61 (sensitivity 62%, specificity 68%, and positive predictive value (PPV) 80%).

#### EVLWI

**Figure 2:**
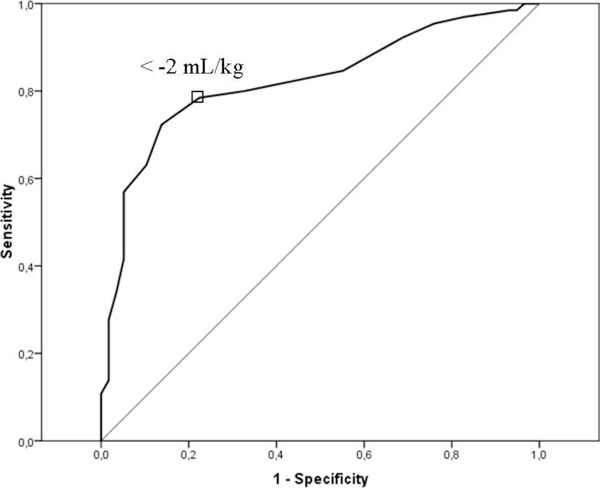
**Receiver-operating characteristic (ROC) curve**. Sensitivity and specificity of Δ_max_EVLWI with respect to 28-day mortality according to ROC analysis in 123 patients. The Area under the curve (AUC) was 0.822.

EVLWI measurements are outlined in Table 2. ROC statistics using baseline EVLWI, EVLWI_max_, and EVLWI_mean _to predict outcome revealed an AUC of 0.513, 0.591, and 0.595, respectively. The best predictor for mortality was EVLWI_max _with a cutoff point of >11 mL/kg, showing a 60% sensitivity and a 57% specificity with a PPV of 61%. EVLWI_max_>11 mL/kg was correlated with a higher percentage of ALI (70% vs 34%, *p *< 0.001), higher tidal volumes (8.8 ± 1.9 vs 7.8 ± 1.4 mL/kg, *p *= 0.001), and a trend to higher mortality (61% vs 44%, *p *= 0.061). Δ_max_EVLWI was significantly lower if CLFM was achieved (−2.4 ± 4.8 vs 1.0 ± 5.5 mL/kg, *p *= 0.001) and in survivors (Table 2). The AUC for Δ_max_EVLWI to predict survival was 0.822. The best cutoff point for Δ_max_EVLWI predicting good outcome was <−2 mL/kg showing a sensitivity of 74% and a specificity of 78% with a PPV of 75% (Figure 2).

**Table 2:** Analysis of EVLWI

Variable	Survivors (*n *= 58)	Nonsurvivors (*n *= 65)	*p *value
EVLWI_min _(mL/kg)	7.3 ± 2.7	8.5 ± 4.1	0.059
EVLWI_max _(mL/kg)	11.7 ± 4.3	13.7 ± 5.9	0.041
EVLWI_mean _(mL/kg)	9.2 ± 3.3	10.7 ± 4.6	0.043
Day EVLWI_max _(day)	2.4 ± 1.4	3.1 ± 2.2	0.026
ΔEVLWI (mL/kg)	−1.3 ± 3.5	2.1 ± 5.0	<0.001
Δ_max_EVLWI (mL/kg)	−3.3 ± 3.8	2.5 ± 5.3	<0.001

EVLWI_min_, minimal EVLWI during ICU stay; EVLWI_max_, maximal EVLWI during ICU stay; EVLWI_mean_, mean EVLWI during ICU stay; ΔEVLWI, difference between first and last extravascular lung water index; Δ_max_EVLWI, maximal difference between extravascular lung water index.

#### IAP measurements

IAP_mean _was lower if CLFM was achieved (8.1 ± 2.6 vs 9.6 ± 3.0 mmHg, *p *= 0.013) and APP on day 3 was significantly higher in survivors (80.7 ± 10.7 vs 70.9 ± 13.5 mmHg, *p *< 0.001). IAH occurred in 25 patients (20%) and was not correlated with 28-day mortality (*p *= 0.658), CLFM achievement (*p *= 0.150), or whether patients were responders or not (*p *= 0.822). Pertinent variables recorded 1 week after ICU admission in the remaining 85 patients are summarized in Table 3.

**Table 3:** Analysis by IAH

Variable (1 week after ICU admission)	No IAH (*n *= 64)	IAH (*n *= 21)	*p *value
SOFA score			
Respiratory	1.5 ± 1.5	1.7 ± 1.8	0.374
Nervous	2.4 ± 1.6	3.5 ± 1.0	0.004
Cardiovascular	2.0 ± 1.6	2.7 ± 1.3	0.092
Liver	0.6 ± 1.0	1.2 ± 1.4	0.038
Coagulation	0.8 ± 1.1	1.3 ± 1.1	0.084
Renal	1.1 ± 1.5	2.4 ± 1.8	0.002
Total	8.3 ± 4.9	12.8 ± 4.9	0.001

Respiratory variables			
Tidal volume (mL/kg of PBW)	8.9 ± 2	8.4 ± 2.3	0.343
Plateau pressure (cmH_2_O)	24.4 ± 6.9	29.1 ± 6	0.010
PEEP (cmH_2_O)	7.3 ± 2.9	10.2 ± 3.7	0.001
Dynamic compliance (mL/cmH_2_O)	43.9 ± 24.2	38.4 ± 13	0.353
PaO_2_/FIO_2_	275.7 ± 98.4	257.8 ± 106.2	0.486

Ventilator-free days	2.1 ± 2.1	1.4 ± 2.1	0.479

Cumulative fluid balance (mL)	5,943 ± 7,125	10,176 ± 7,523	0.024

EVLWI (mL/kg)	9.8 ± 4.3	9.2 ± 3.7	0.592

IAH, intra-abdominal hypertension; SOFA, sequential organ failure assessment; PBW, predicted body weight; PEEP, positive end-expiratory pressure; EVLWI, extravascular lung water index.

#### Cumulative fluid balance

**Figure 3:**
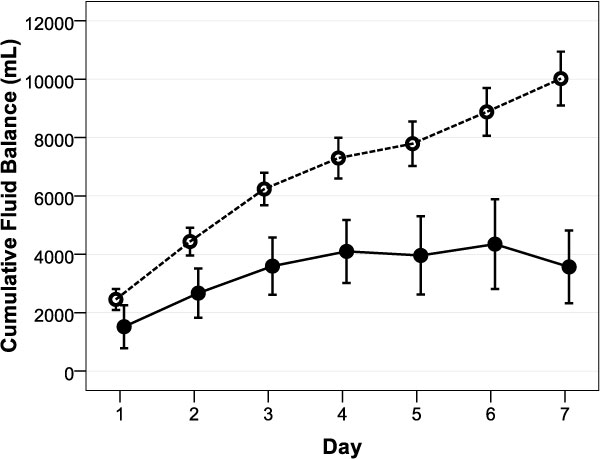
**Evolution of cumulative fluid balance in (non)responders**. Mean ± standard error cumulative fluid balance for the first week after ICU admission. Responders are depicted by a full line and nonresponders by a dotted line. *p < 0.05, day-by-day pairwise compared between responders and nonresponders (one-way ANOVA).

Cumulative fluid balance after 1 week was significantly lower in survivors (4,970 ± 7,737 vs 9,502 ± 6,909 mL, *p *= 0.008), patients achieving CLFM (1,056 ± 7,047 vs 10,282 ± 5,788 mL, *p *< 0.001), and responders (3,567 ± 7,984 vs 10,021 ± 5,920 mL, *p *< 0.001) as shown in Figure 3.

#### Total SOFA score

Total SOFA score remained significantly lower on each day from day 2 in survivors, patients achieving CLFM, and responders (*p *< 0.001).

### Clinical outcomes

**Figure 4:**
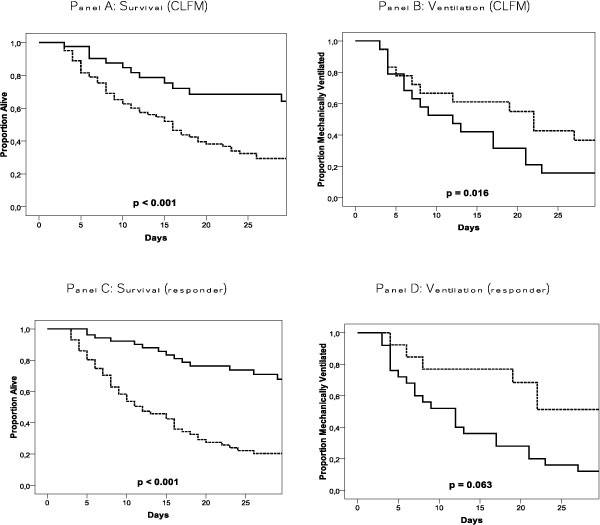
**Kaplan-Meier plots**. Kaplan-Meier plots for cumulative survival and proportion of patients on MV. We compared CLFM and no CLFM achievement (full lines and dotted lines, respectively) in A (survival) and B (ventilation). In C (survival) and D (ventilation), responders and nonresponders were compared (full lines and dotted lines, respectively).

Outcomes concerning organ function were described by the course of total SOFA score as above. Other major outcomes are shown in Table 4 and Kaplan-Meier plots are shown in Figure 4.

**Table 4:** Major outcome variables

			Responder (*n *= 52)	Nonresponder (*n *= 71)	*p *value
First week	Organ-failure-free days	Respiratory	5.5 ± 1.9	3.9 ± 2.4	<0.001
		Nervous	2.1 ± 2.6	1.7 ± 2.2	0.454
		Cardiovascular	3.4 ± 2.7	1.4 ± 2.1	<0.001
		Liver	6.1 ± 1.9	5.3 ± 2.3	0.046
		Coagulation	5.9 ± 2.1	5.0 ± 2.4	0.031
		Renal	4.8 ± 2.7	3.9 ± 2.7	0.063
	
	Ventilator-free days		2.5 ± 2.3	1.5 ± 2.3	0.023

First 28 days	Death (%)		25.0	73.2	<0.001

Mortality and duration of MV were lower in patients achieving CLFM and in responders. Responders had fewer days with cardiovascular, respiratory, liver, and coagulation failure during the first week of ICU admission.

Multivariate analysis identified that increasing IAP_mean _and CLI on day 3 and being a nonresponder were independent risk factors for not achieving CLFM (*p *= 0.919 Hosmer-Lemeshow test) (Table 5). Increasing baseline creatinine and EVLWI_max_, decreasing APP on day 3, not achieving CLFM, and being a nonresponder were independent risk factors for 28-day mortality (*p *= 0.808 Hosmer-Lemeshow test) (Table 6).

**Table 5:** Multivariate analysis of independent risk factors for not achieving CLFM

	Variable	Adjusted OR	95% CI	*p *value
**Baseline**	Age (years)	1.00	0.97-1.03	0.832
	BMI (kg/m^2^)	0.93	0.85-1.01	0.073

**Day 3**	Total SOFA score	1.03	0.92-1.16	0.575
	CLI >61	2.76	1.02-7.48	0.046

**ICU stay**	IAP_mean _(mmHg)	1.28	1.06-1.54	0.011
	Nonresponder	5.52	2.01-15.15	0.001

OR, odds ratio; CI, confidence interval; BMI, body mass index; CLI, capillary leak index; IAP, intra-abdominal pressure.

**Table 6:** Multivariate analysis of independent risk factors for 28-day mortality

	Variable	Adjusted OR	95% CI	*p *value
**Baseline**	Age (years)	1.01	0.96-1.05	0.801
	BMI (kg/m^2^)	0.92	0.83-1.03	0.142
	Creatinine (mg/dL)	1.89	1.03-3.48	0.041

**Day 3**	APP (per −10 mmHg)	2.20	1.25-3.89	0.007
	Total SOFA score	1.01	0.89-1.15	0.852

**ICU stay**	EVLWI_max _>11 mL/kg	4.57	1.32-15.63	0.016
	CLFM not achieved	9.34	2.39-36.93	0.001
	Nonresponder	7.14	2.23-22.91	0.001

OR, odds ratio; CI, confidence interval; BMI, body mass index; APP, abdominal perfusion pressure; SOFA, sequential organ failure assessment; EVLWI_max_, maximal EVLWI during ICU stay; CLFM, conservative late fluid management.

## Discussion

**Figure 5:**
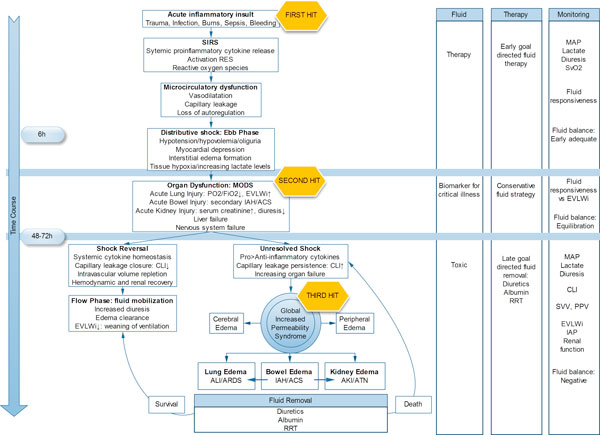
**Proposed time course in shock, introducing a three-hit model and global increased permeability syndrome**.

Our study demonstrated that a persistent increase in CLI, EVLWI, and fluid balance in critically ill patients is associated with poor outcome. We investigated the precise prognostic value of these parameters and were able to formulate a unifying hypothesis implementing concepts of earlier studies (Figure 5).

As early as 1942, Cuthbertson introduced the concept of a dual metabolic response to bodily injury [30]. In direct response to initial proinflammatory cytokines and stress hormones, the ebb phase represents a distributive shock characterized by arterial vasodilatation and transcapillary albumin leak [31] abating plasma oncotic pressure. Arterial underfilling, microcirculatory dysfunction, and secondary interstitial edema lead to systemic hypoperfusion and impaired regional tissue oxygenation [2]. In this early stage of shock, adequate fluid therapy comprises of goal-directed filling [3] to prevent evolution to multiple organ dysfunction syndrome (MODS). As compensatory neuroendocrine reflexes and potential renal dysfunction result in sodium and water retention [32], positive fluid balances are inherent in the ebb phase. Patients with higher severity of illness need more fluids to achieve cardiovascular optimization. Therefore, at this point, fluid balance may be considered a biomarker of critical illness [33].

Patients overcoming shock attain homeostasis inflammatory mediators within 3 days [1]. Subsequent hemodynamic stabilization and restoration of plasma oncotic pressure set off the flow phase with resumption of diuresis and mobilization of extravascular fluid resulting in negative fluid balances. In line with Murphy et al. [12], we found CLFM achievement to be a strong and independent predictor of survival. In contrast, patients with persistent systemic inflammation maintain capillary leak and do not reach the flow phase, accumulating further positive fluid balances. In this context, we introduce the global increased permeability syndrome (GIPS), characterized by nonresponders with increased CLI, no CLFM achievement, and progressing organ failure. GIPS represents a 'third hit' of shock following acute injury and MODS.

We defined CLI as a parameter of capillary leak, assuming that increased vascular permeability caused by systemic inflammation is associated with high CRP levels [34] and hypoalbuminemia [31]. CLI had a biphasic course and the maximum reached on the third day of shock was an independent predictor of CLFM achievement. Previously, a negative cumulative balance [8,12,13,35,36] and lower PVPI [22] on day 3 were correlated with better survival. The third day of shock seems to be a crucial turning point [37] at which homeostasis of cytokines is accompanied by the healing of microcirculatory disruptions and 'closure' of the capillary leak. This interpretation is supported by Boerma et al. who demonstrated normalization of the microcirculatory blood flow on day 3 in septic patients [38].

As a result of capillary leak and an impaired flow phase, overzealous administration of fluids in GIPS will lead to gross fluid overload and tissue edema [14]. Interstitial edema raises the pressure in all four major body compartments: head, chest, abdomen, and extremities. Consequently, venous resistance of organs within compartments increases and perfusion pressure decreases contributing to progression of organ failure. As different compartments interact and reciprocally transmit compartment pressures, the concept of polycompartment syndrome is suggested [39].

The abdomen plays a central role in GIPS and polycompartment syndrome. Positive cumulative fluid balance is a known risk factor for secondary IAH [40] which in turn is associated with renal dysfunction [41]. Therefore, fluid overload leading to IAH and renal dysfunction may counteract its own resolution. Data from our study support these ideas, demonstrating higher average positive cumulative fluid balance and renal SOFA score after 1 week in patients developing IAH. Moreover, we determined increased IAP_mean _as an independent risk factor for no CLFM achievement and decreased APP as risk factor for 28-day mortality.

As the adverse effects of fluid overload in states of capillary leak are particularly pronounced in the lungs [17], monitoring EVLWI may offer a valuable tool to guide fluid management in the critically ill. In line with previous reports, we established a correlation between EVLWI_max _during admission and poor outcome [42]. An increased EVLWI_max _may indicate a state of capillary leak, associated with a higher severity of illness and mortality [11,22,23,42]. In this context, data from Sturm et al. are particularly of interest, correlating EVLWI with albumin extravasation in patients after multiple trauma [43].

The course of EVLWI during the first week of admission may even be a better outcome predictor. Responders, defined as patients with an EVLWI decrease of >2 mL/kg, were more likely to achieve CLFM, had more organ-failure-free and ventilator-free days, and a better 28-day outcome. These data suggest that responders overcome the distributive shock and make a transition to the flow phase. Nonresponders on the other hand stay in the grip of the ebb phase and progress to GIPS associated with interstitial fluid accumulation, organ failure, and death. In this hypothesis, (the change in) EVLWI has a prognostic value as a reflection of the extent of capillary leak rather than as a quantification of lung function impairment. Indeed, the degree of hypoxemia in ARDS is an inferior prognostic factor, as extrapulmonary organ failure mostly determines outcome [44]. Accordingly, in a subgroup analysis of patients with ARDS, Sakka et al. found no higher maximum EVLWI in nonsurvivors [42]. Therefore, in an established state of capillary leak, time-dependent changes in EVLWI appear to be of superior value.

Our observations may have direct consequences on fluid management in the critically ill. Patients at risk for GIPS require restrictive fluid strategies and even fluid removal to avoid interstitial edema formation.

Our study has several important limitations. First, the observational nature of this study does not allow discrimination between a primary and secondary effect of fluid balance on outcome; prospective trials are warranted to determine if fluid overload is cause or consequence of worse outcome. Second, inclusion of patients was based on clinical appraisal of the need of MV and thermodilution catheter monitoring. Therefore, the studied population was a specific case mix of seriously ill patients selected without well-defined objective rules making simple extrapolation of our results to a general ICU population impossible. However, albeit in this particular population, our observations contributed to some basic ideas regarding fluid management in patients with capillary leak as proposed in earlier reports [1,14,16,37,40] and raised questions that should be addressed in future prospective investigations. Third, our database did not supply detailed information on the amounts of fluids administrated specified for the first 6 h. Early fluid resuscitation has an important impact on outcome [12,45]. There were no data on the type of fluids and infusion rates used during ICU stay either. Fourth, differences in MAP and GEF at baseline may be important confounding factors as they may reflect different hemodynamic states dictating whether a patient can mobilize fluids in the flow phase.

## Conclusions

We identified a subgroup of mechanically ventilated patients with persistent capillary leak failing to reach the flow phase. In these patients, GIPS may reflect a 'third hit' and superfluous fluid administration may be considered toxic. Future prospective clinical trials evaluating any therapy aimed at a reduction of EVLWI are warranted.

## Abbreviations

ALI: acute lung injury; APP: abdominal perfusion pressure; AUC: area under the curve; CLFM: conservative late fluid management; CLI: capillary leak index; CO: cardiac output; Day_max_: day on which maximal EVLWI was measured; EVLW(I): extravascular lung water (index); ΔEVLWI: difference between first and last extravascular lung water index; Δ_max_EVLWI: maximal difference between extravascular lung water index; GEDV(I): global end-diastolic volume (index); GEF: global ejection fraction; GIPS: global increased permeability syndrome; IAH: intra-abdominal hypertension; IAP: intra-abdominal pressure; ICU: intensive care unit; MAP: mean arterial pressure; MV: mechanically ventilated; PPV: positive predictive value; PVPI: pulmonary vascular permeability index; ROC: receiver-operating characteristics; SOFA: sequential organ failure assessment; vs: versus.

## Competing interests

WH and MM are members of the medical advisory board of Pulsion Medical Systems (Munich, Germany), a monitoring company. The other authors declare that they have no competing interests.

## Authors' contributions

CC, IDL, NVR, KS, HD, and MM planned the study and were responsible for the design, coordination, and drafting the manuscript. WH participated in the study design and helped to draft the manuscript. CC and MM performed the statistical analysis and helped to draft the manuscript. All authors read and approved the final manuscript.
